# A Novel Function of Mitochondrial Phosphoenolpyruvate Carboxykinase as a Regulator of Inflammatory Response in Kupffer Cells

**DOI:** 10.3389/fcell.2021.726931

**Published:** 2021-12-14

**Authors:** Haibo Dong, Yue Feng, Yang Yang, Yun Hu, Yimin Jia, Shu Yang, Nannan Zhao, Ruqian Zhao

**Affiliations:** ^1^ MOE Joint International Research Laboratory of Animal Health and Food Safety, Nanjing Agricultural University, Nanjing, China; ^2^ Key Laboratory of Animal Physiology and Biochemistry, Nanjing Agricultural University, Nanjing, China

**Keywords:** inflammation, Pck2, protein phosphorylation, Kupffer cell, mitochondrial dysfunction

## Abstract

**Background:** There has been a recent appreciation that some metabolic enzymes can profoundly influence the nature of the immune response produced in macrophages. However, the role of mitochondrial phosphoenolpyruvate carboxykinase (PCK2) in immune response remains unknown. This study aims to investigate the role of PCK2 in lipopolysaccharides (LPS)-induced activation in Kupffer cells.

**Methods:** Inflammatory cytokines were determined by real-time quantitative reverse transcription-polymerase chain action (qRT-PCR) and flow cytometric analysis using a cytometric bead array. Western blotting and immunofluorescence staining were used to determine PCK2 expression and subcellular distribution under confocal laser microscopy. qRT-PCR, flow cytometry, and high-performance liquid chromatography (HPLC) were used to determine mitochondrial function. Pharmacological inhibition, knockdown, and overexpression of PCK2 were used to confirm its function. Co-immunoprecipitation (Co-IP) was performed to determine MAPK/NF-κB phosphorylation.

**Results:** Inflammatory response was significantly increased in LPS-treated mice and Kupffer cells. During the inflammatory process, the protein level of PCK2 was significantly upregulated in Kupffer cells. Interestingly, the localization of PCK2 was mainly in cytosol rather than mitochondria after LPS stimulation. Gain-of-function and loss-of-function analyses found that PCK2 overexpression significantly upregulated the levels of inflammation markers, whereas PCK2 knockdown or inhibition significantly mitigated LPS-induced inflammatory response in Kupffer cells. Furthermore, PCK2 promoted protein phosphorylation of NF-κB and AKT/MAPK, the major signaling pathways, controlling inflammatory cascade activation.

**Conclusion:** We identified a novel function of PCK2 in mediating LPS-induced inflammation and provided mechanistic insights into the regulation of inflammatory response in Kupffer cells. Therefore, PCK2 may serve as a novel therapeutic target for the regulation of Kupffer cells-mediated inflammatory responses.

## Introduction

Inflammation is a biological response when the immune system is activated by a variety of factors, including pathogens, damaged cells, and toxic compounds (Chen et al., 2018). Lipopolysaccharide (LPS) is the major outer surface membrane component of Gram-negative bacteria. As a pathogen-associated molecular pattern (PAMP), circulating LPS induces both local or systemic inflammatory responses (Mogensen, 2009). Kupffer cells are the most abundant population of resident tissue macrophage in the body and act as an important modulator for the initiation, maintenance, and outcome of liver inflammation (Kolios et al., 2006; Wynn et al., 2013). Kupffer cells are located in the lumen of the liver sinusoids that facilitate exposure to gut-derived bacteria, microbial debris, and bacterial endotoxins (Wynn et al., 2013). Accumulating evidence showed that the interaction between Kupffer cells and LPS may be the initiating event leading to hepatotoxicity in different types of liver injuries including endotoxemia, alcoholic liver injury, systemic viral infections, tumor initiation, progression, and metastasis (Kolios et al., 2006; Kitagawa et al., 2013; Li et al., 2014; Shulga and Pastorino, 2016). Thus, further investigation of the underlying mechanisms in the response of Kupffer cells to inflammatory stimulation is essential to develop effective therapeutic strategies for liver injury.

Metabolic shifts are important features of macrophage immune activation (Pearce and Pearce, 2013). In response to pathogen-derived signals and cytokines, immune cells undergo metabolic reprogramming (Jing et al., 2020). Correspondingly, the changes in cellular metabolism also profoundly influence the nature of the immune response. For example, LPS and/or IFN-γ-activated macrophages, known as M1 macrophages (pro-inflammatory phenotype) undergo an increase in glycolysis but a reduction in Krebs cycle-associated oxidative phosphorylation (OXPHOS) (Odegaard and Chawla, 2011; Galvan-Pena and O’neill, 2014; Orecchioni et al., 2019), whereas macrophages activated by IL-4 stimulation, known as M2 macrophages (anti-inflammatory phenotype), yield an induction of OXPHOS (Bi et al., 2019). In addition, growing evidence indicates that metabolic enzymes play a key role in immune activation. For example, hexokinase Ⅱ (HK- Ⅱ) is an enzyme that phosphorylates glucose into glucose 6-phosphate (G6P) in the glycolytic pathway. It is strongly associated with activation, proliferation, and inflammatory response in various cell types including T cells, DCs, and macrophages (Seki and Gaultier, 2017). Recent observations revealed that α-enolase (ENO-1) is another glycolytic enzyme, presents a non-catalytic function depending on its subcellular localization. In macrophages, LPS stimulation induced ENO-1 translocation from the cytosol to the plasma membrane (Bae et al., 2012). All of these indicate that the metabolic enzymes and their subcellular localization are very important for immune cell function.

Phosphoenolpyruvate carboxykinase (PEPCK) is the rate-limiting enzyme of gluconeogenesis (Odegaard and Chawla, 2013), which is synthesized as two distinct isoforms, a cytosolic isoform (PCK1, PEPCK-C) and a mitochondrial isoform (PCK2, PEPCK-M). The activity of PEPCK is present mostly in the liver, but also in some non-gluconeogenic tissues and cells including brown/white adipose tissue and lymphocytes (Warburg et al., 1927). As a kinase, PCK2 converts oxaloacetate (OAA) to phosphoenolpyruvate (PEP), which serves as a bridge between glycolytic and TCA cycle intermediates in mitochondria (Warburg et al., 1927; Modaressi et al., 1998). Recent studies have demonstrated that PCK2 is important in diabetes and obesity development, as well as tumor cell adaptation (Beale et al., 2007; Mendez-Lucas et al., 2014; Leithner, 2015; Leithner et al., 2015; Vincent et al., 2015). However, the role of PCK2 in the inflammatory response is unknown. Thus, this study was aimed to investigate the role of PCK2 in regulating Kupffer cell immune activation.

## Materials and Methods

### Mice

Mice used in the study were obtained from Jiangning District Qinglongshan Animal Breeding Farms, Nanjing, China. Eight-week-old C57BL/6J male mice were divided into two groups of six mice each. Saline-treated mice were served as the control group (Ctrl). LPS-treated mice were served as treatment group (LPS), which received intravenous injections of LPS (dissolved in sterile saline) at a dose of 5 mg/kg body weight to induce acute inflammation based on a previous publication (Sadasivam et al., 2014). After 2 h, blood samples were taken, and plasma was separated and stored at −20°C. Liver (without the gall bladder) samples were rapidly frozen in liquid nitrogen and kept at −80°C for further analysis.

### Isolation of Primary Mouse Hepatocyte and Treatment

A two-step perfusion method was used and modified (Lee et al., 2013). In this procedure, the liver of C57BL/6J male mice was perfused with a Ca2^+^ free buffer (140 mmol/L NaCl, 6.7 mmol/L KCl, 10 mmol/L HEPES, 2.5 mmol/L glucose, 0.5 mmol/L EDTA, pH = 7.4) followed by perfusion with a collagenase buffer containing Ca^2+^ (140 mmol/L NaCl, 6.7 mmol/L KCl, 30 mmol/L HEPES, 2.5 mmol/L glucose, 5 mmol/L CaCl2, pH = 7.4). The collagenase type Ⅳwas used at 200 μg/ml (Sigma-Aldrich, St. Louis, MO). The removal of Ca^2+^ in the first step helps to disrupt desmosomes, while the addition of Ca2^+^ in the second step is required for optimum collagenase activity. Primary hepatocytes were seeded at 2 × 10^5^ cells/well in a 6-well tissue culture plate for 24 h, then cells were treated with or without *Escherichia coli (O111:B4)*-derived lipopolysaccharide (LPS; Sigma-Aldrich St. Louis, MO, United States) at 1 μg/ml for 12 h.

### Inflammatory Treatment and Cell Transfection

Kupffer cell line was obtained from BeNa Culture Collection (BNCC340733). Cells were cultured in RPMI medium supplemented with 10% fetal bovine serum (FBS), 10,000 units/mL penicillin, 10 mg/ml streptomycin and incubated in a humidified atmosphere of 5% CO_2_ at 37°C. *Escherichia coli* (*O111:B4*)-derived lipopolysaccharide (LPS; Sigma-Aldrich St. Louis, MO, United States) was used to create an inflammatory microenvironment *in vitro*. Kupffer cells were treated with LPS (1 μg/ml) for 12 h and/or 24 h. PCK2 inhibitor 3-mercaptopicolinic acid, hydrochloride (3MP, sc-206655) was obtained from Santa Cruz Biotechnology. Transient knockdown of PCK2 was achieved using siRNA (CCG​CAT​TAT​GTA​TGT​GCT​T) ordered from Guangzhou RiboBio Co. Ltd. The full-length PCK2 (Gene Bank accession number: NM_028994.3) cDNA was cloned into the pcDNA3.1 vector. Lipofectamine 3,000 was used as the transfection reagent. When cells reached 80% confluency, siRNA or control plasmids were transfected into Kupffer cells for 24 h according to the manufacturer’s instructions.

### Histology and Periodic Acid-Schiff Staining in Liver Tissues

Excised liver tissues were rinsed with cold PBS, fixed overnight in 4% paraformaldehyde for paraffin embedding, and 5 µm sections were cut. For histology, liver tissue slides were stained with hematoxylin and eosin (H and E staining). Hepatic glycogen was detected using a PAS staining kit (C0142S, Beyotime Biotechnology, Shanghai, China).

### Mitochondrial Phosphoenolpyruvate Carboxykinase2 Enzymatic-Activity Assay

Enzymatic activities of PCK2 were measured with a PEPCK activity kit (A131-one to one, Nanjing Jian Cheng Bioengineering Institute, Nanjing, China) according to the manufacturer’s instructions.

### Immunofluorescence Staining

The prepared cells were washed twice with PBS and fixed for 15 min at 4°C using 4% paraformaldehyde. The cells were washed again with PBS, incubated for 20 min at 4°C in PBS that contained 0.3% Triton X-100, and then reactions were induced for 1 h at room temperature in PBS containing 2% bovine serum albumin (BSA) to suppress non-specific reactions. The anti-NF-κB p65 antibody was then diluted to 1:200 in a PBS solution that contained 2% BSA and incubated for 2 h at room temperature. The cells were then washed three times in PBS and fluorescein isothiocyanate (FITC)-conjugated IgG (Molecular Probes, Eugene, OR, United States), which is a secondary antibody, was diluted to 1:100 and incubated for 1 h at room temperature. Samples of the immunofluorescence-stained cells were observed under a confocal laser scanning microscope (Olympus, Tokyo, Japan). A wavelength of 488 nm was used for FITC, and the images were reassembled into final three-dimensional images according to the manufacturer’s instructions (Olympus Fluoview 300, Olympus).

### Cytometric Bead Array Assay for Cytokines

The plasma was harvested from mice after 2 h LPS treatment, the cell culture supernatant was collected after 12 h LPS treatment. All treated samples were subjected to BD CBA Mouse Flex Set (BD Biosciences, Franklin Lakes, NJ, United States) according to the manufacturer’s protocol for cytokine measurement. Capture beads coated with antibodies against six different cytokines: IL-6, IL-10, MCP1, IL-12p70, TNF-α, and IFN-γ were incubated in each sample. PE-conjugated antibodies were added for the detection of cytokines, while cytokine quantification was based on a standard curve plot. Fluorescence from PE-conjugated antibodies was detected using BD Accuri™ C6 Plus flow cytometer (BD Biosciences, Franklin Lakes, NJ, United States) and NovoCyte™ flow cytometer (ACEA Biosciences, San Diego, CA, United States) during sample data acquisition, while data analyses were performed using FCAP Array Software v3.0 (BD Biosciences, Franklin Lakes, NJ, United States) and NovoExpress® Software v1.2.4 (ACEA Biosciences, San Diego, CA, United States) respectively.

### Mitochondrial Membrane Potential Detection

Mitochondrial membrane potential was detected using the JC-1 Mitochondrial Membrane Potential Assay Kit (40706ES60, Yisheng Biotechnology Co., Ltd., Shanghai, China), according to the manufacturer’s protocol. Briefly, following treatment, the cell culture medium was removed, and cells were resuspended in 0.5 ml of JC-1 staining solution and then incubated at 37°C for 20 min in a light-proof cell incubator. Then, cells were centrifuged at 4°C for 3 min at 600 × *g*, the supernatant was discarded, cells were washed twice with the JC-1 dye buffer, and cells were resuspended. FITC and PE fluorescence readings were used as a measure of mitochondrial membrane potential. FITC fluorescence was detected at 488 and 530 nm by flow cytometry, while the PE fluorescence signal was excited at 530 nm and detected at 630 nm.

### High-Performance Liquid Chromatography Assay

HPLC was performed according to a previous study with minor modification (Shurubor et al., 2016). Briefly, Agilent Eclipse XDB-C18 column was used as the solid phase (4.6 mm × 150 mm, 5 μm) The chromatographic conditions were as follows: UV detector wavelength was at 210 nm; mobile phase was methanol: 0.01 mol/L KH_2_PO_4_-H_3_PO_4_ buffer solutions (3∶ 97, pH = 2.85); flow rate was 0.8 ml/min; column temperature was 25°C; sampling volume was 20 μl.

### Oxygen Consumption Rate Measurement

The process was performed according to the manufacturer’s protocol. Briefly, Kupffer cells were plated on a 24-well cell culture microplate (Agilent Technologies, United States) overnight. Oxygen consumption rates (OCR) was measured using Seahorse XF Cell Mito Stress Test Kit (103015-100, Agilent Technologies, United States) by Agilent Seahorse XFe24 Analyzers, the cells were incubated in XF assay medium (Seahorse XF-RPMI containing either 1 mM sodium pyruvate, 2 mM l-glutamine and 10 mM glucose). Three measurements were obtained under basal conditions and upon addition of oligomycin (1.5 μM), fluoro-carbonyl cyanide phenylhydrazone (FCCP, 0.5 μM), and rotenone/antimycin A (0.5 μM).

### Nuclear, Cytosolic, and Mitochondrial Protein Extraction

Kupffer cells were cultured in a 10cm dish, then treated with LPS and harvested at 12 h. The nuclear protein fractions were prepared using a nuclear and cytoplasmic protein extraction kit (Beyotime Biotech Inc., Nantong, Jiangsu, China) according to the manufacturer’s protocol. The cytosolic and mitochondrial protein fractions were prepared using cell mitochondria isolation kit (Beyotime Biotech Inc., Nantong, Jiangsu, China) according to the manufacturer’s protocol.

### Total RNA Isolation and Real-Time PCR

Total RNA was isolated from Kupffer cells using TRIzol Reagent (Invitrogen, United States) and then treated with RNase-free DNase and reverse-transcribed to cDNA using random hexamer primers (Promega, United States). Two microliters of diluted cDNA (1:20, vol/vol) were used for real-time PCR with Mx3000 P Real-Time PCR System (Stratagene, United States). All primers (Supplementary Table S1) were synthesized by Generay Biotech (Shanghai, China). Several reference genes were tested and ribosomal protein S17 (RPS17) was chosen as a reference gene. Data were analyzed using the method of 2^−ΔΔCT^.

### Total Protein Extraction and Western Blotting

Cells were homogenized in radioimmunoprecipitation assay buffer supplemented with protease and phosphatase inhibitors and centrifuged at 15,000 g for 15 min at 4°C. The protein concentration was determined according to the manufacturer’s instructions of the Pierce BCA Protein Assay kit (Rockford, IL, United States). Western blots were performed with 20–50 μg of protein. Proteins were separated in 8–10% SDS-PAGE and transferred to a nitrocellulose membrane. Western blot analysis for TLR2 (SC10739, Santa Cruz, United Kingdom, diluted 1:500), TLR4 (SC30002, Santa Cruz, United Kingdom, diluted 1:1,000), NFκB (ab16502, Abcam, United Kingdom, diluted 1:1,000), p-p65 (ab86299, Abcam, United Kingdom, diluted 1:1,000), PCK2 (ab70359, Abcam, United Kingdom, diluted 1:1,000), β-actin (AP0060, Bioworld Technology, United States, diluted 1:10,000), AKT (BS1007, Bioworld Technology, United States, diluted 1:1,000), mitochondrial transcription factor A (TFAM) (BS7319, Bioworld Technology, United States, diluted 1:1,000), COX Ⅳ (AP0707, Bioworld Technology, United States, diluted 1:1,000) were carried out according to the recommended protocols provided by the manufacturers. Images were captured by Versa Doc 4000MP system (Bio-Rad, United States) and the band density was analyzed with Quantity One software (Bio-Rad, United States).

### Statistical Analysis

All data are expressed as mean ± SEM. The differences between groups were analyzed using Student’s t-test or two-way ANOVA followed by Tukey’s test for multiple comparisons with SPSS 20.0. The differences were considered statistically significant when *p* < 0.05.

## Results

### Mitochondrial Phosphoenolpyruvate Carboxykinase2 Is Decreased in Hepatocytes but Increased in Kupffer Cells After Acute Lipopolysaccharide Administration in Mice

**FIGURE 1:**
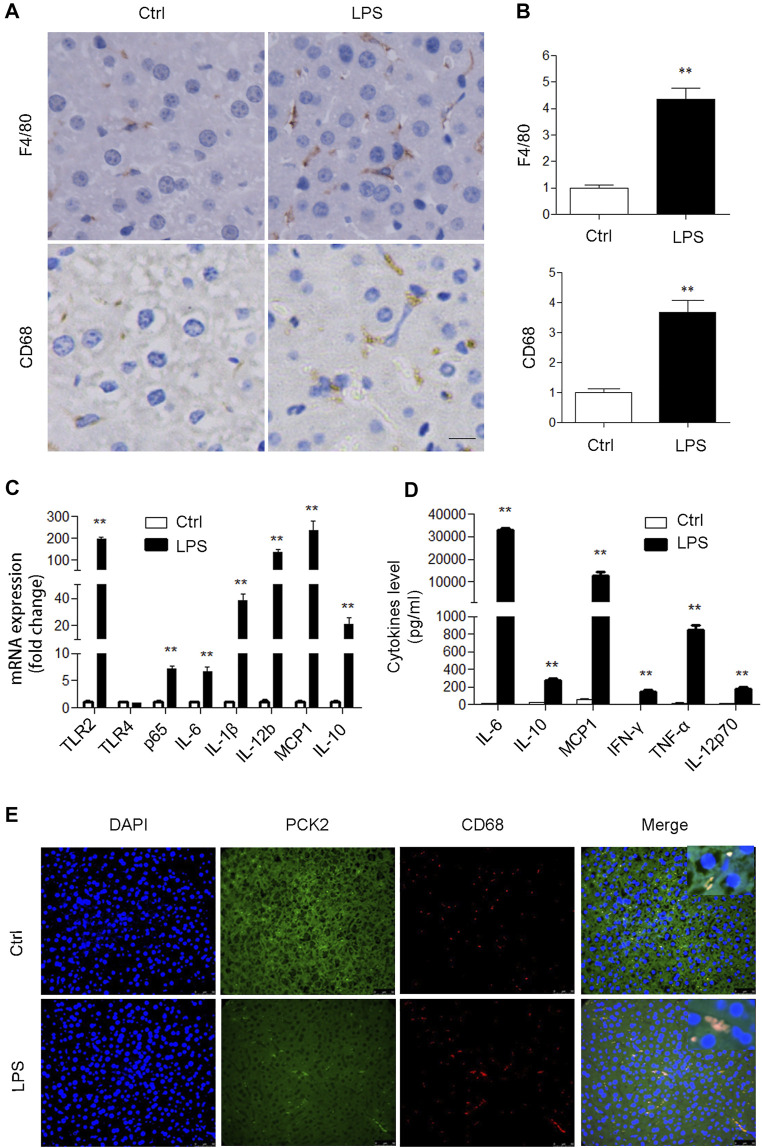
PCK2 is increased in Kupffer cells after acute LPS administration in mice. **(A)** Immunohistochemical staining and quantification of F4/80 and CD68 in the liver (magnification, x400). **(B)** The expression levels of inflammatory cytokines were determined by real-time PCR. **(C)** The levels of inflammatory cytokines in plasma were determined by cytometric bead array (CBA). **(D)** Co-immunofluorescence staining of PCK2 and CD68. Values are mean ± SEM, n = 6. **p < 0.01 versus control group.

To determine if PCK2 is involved in an inflammatory response in the liver, an LPS-induced acute inflammatory mouse model was created. Although LPS administration had no significant effect on body weight, liver weight, and liver index, it increased plasma AST, ALT, and LDH levels, and decreased plasma glucose levels (Supplementary Table S2). H and E staining showed that LPS stimulation damaged hepatic lobule, ruined hepatic cords, and increased the number of infiltrated inflammatory cells (Supplementary Figure S1A). These results indicate the development of liver damage after LPS acute administration. Subsequently, we determined the activation of Kupffer cells in LPS-induced liver injury in mice. F4/80 and CD68 (macrosialin) are commonly used as surface markers to identify liver Kupffer cells (Kinoshita et al., 2010), and an increased expression of F4/80 and CD68 positive Kupffer cells was observed in LPS treated mice (Figures 1A,B). Such increased expression of F4/80 and CD68 could not be ascribed to the Kupffer cells proliferation as LPS stimulation did not increase Kupffer cell numbers *in vitro* (Supplementary Figure S1A,B). Next, the mRNA levels of inflammatory genes and the production of cytokines were determined by qRT-PCR and flow cytometry, respectively. The results showed that TLR2 and its downstream signaling molecules were significantly upregulated in the liver (Figure 1C). Accordingly, serum inflammatory factors including IL-6, IL- 10, MCP1, IFNγ, TNFα, IL10, and IL-12p70 were markedly increased (Figure 1D). We also determined hepatic glycogen content and gluconeogenesis, which reflected the typical metabolic changes during LPS stimulation. PAS staining demonstrated that LPS promoted hepatic glycogen consumption, while Western blot analysis revealed that hepatic gluconeogenic enzymes including PCK1 and PCK2 were suppressed in the liver (Supplementary Figure S2A,B). Moreover, we confirmed that PCK2 was deceased in hepatocytes after LPS treatment by using isolated primary hepatocytes (Supplementary Figure S2C). In addition, we found that PCK1 was not expressed in Kupffer cells (Supplementary Figure S2D). Unlike PCK1, PCK2 was also expressed in Kupffer cells. Immunofluorescence double staining revealed that PCK2 was decreased in hepatocytes but increased in Kupffer cells following LPS treatment (Figure 1E).

**FIGURE 2:**
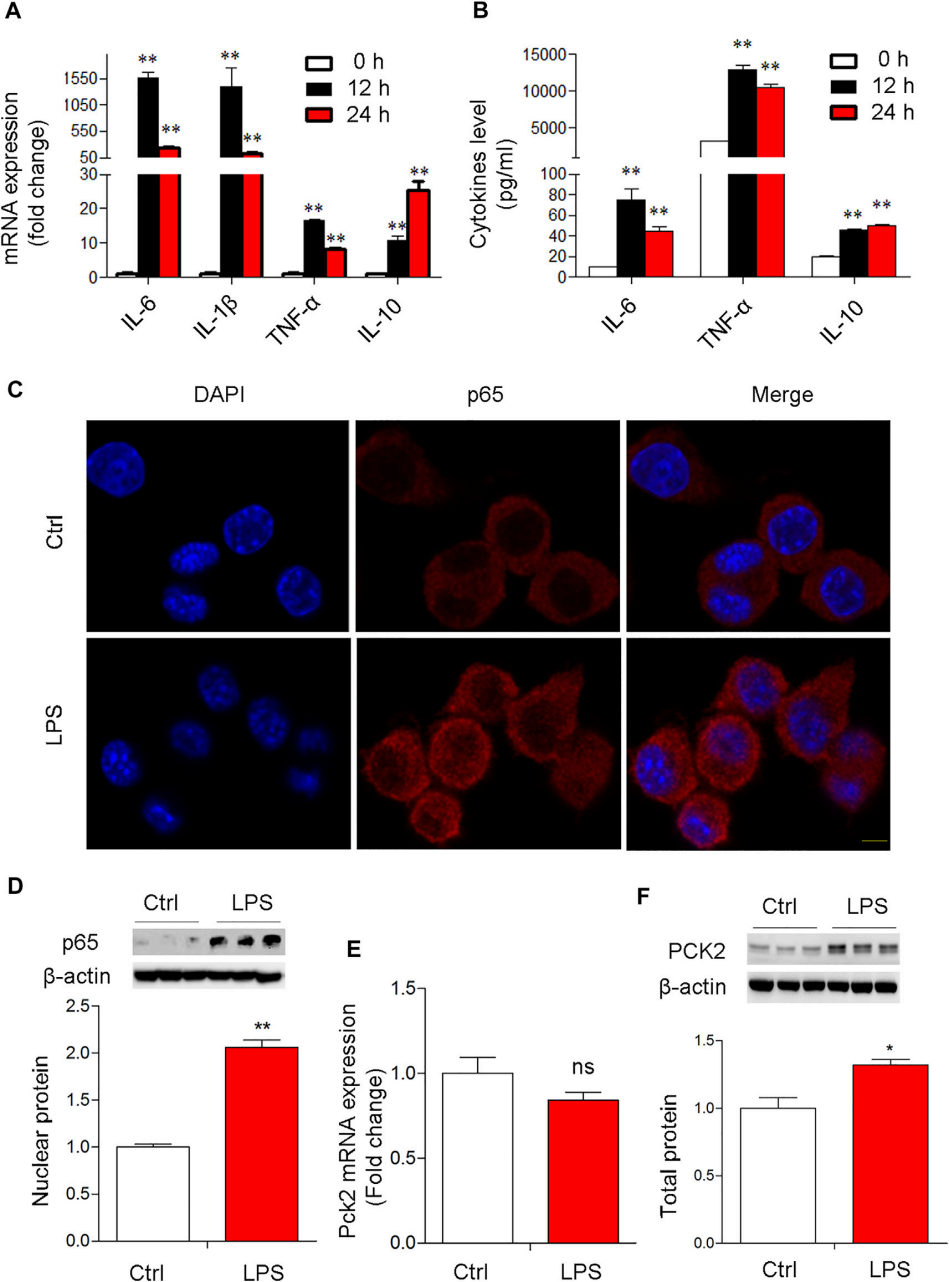
LPS-induced NF-κB activation and PCK2 upregulation *in vitro*. **(A)** The mRNA expression of inflammatory cytokines in LPS-treated Kupffer cells (1 μg/ml) at different time-points. **(B)** The production of IL-6, TNF-α, and IL-10 was measured in the medium by CBA. **(C)** Representative fluorescence images of P65 nuclear translocation in Kupffer cells (scale bar: 20μm). **(D)** Nuclear p65 NF- κB was measured by Western blot. **(E)** The mRNA expression of PCK2 was determined by real-time PCR. **(F)**, The total protein levels of PCK2 were determined by Western blot. Values are mean ± SEM. *p < 0.05, **p < 0.01.

Next, to confirm the expression of PCK2 in Kupffer cells upon LPS stimulation, a mouse cell line of Kupffer cells was used for *in vitro* study. The data showed that the mRNA levels of pro-inflammatory genes in the cells and cytokines production in the medium were significantly increased at 12 and 24 h after LPS stimulation (Figures 2A,B). TLR2 rather than TLR4 was up-regulated after LPS stimulation (Supplementary Figure S3A,B). Consequently, p65 NF-κB nuclear translocation was observed by Immunofluorescence microscopy and Western blot (Figures 2C,D). These data indicate that LPS induced a strong inflammatory response in Kupffer cells. Then, PCK2 expression was determined by real-time PCR and Western blot. We found that the upregulation of PCK2 was only at its protein level while its mRNA expression was not impacted by LPS stimulation (Figures 2E,F).

### Lipopolysaccharide-Induced Mitochondrial Dysfunction and Mitochondrial Phosphoenolpyruvate Carboxykinase2 Aggregates in Cytosol but Not Mitochondria

**FIGURE 3:**
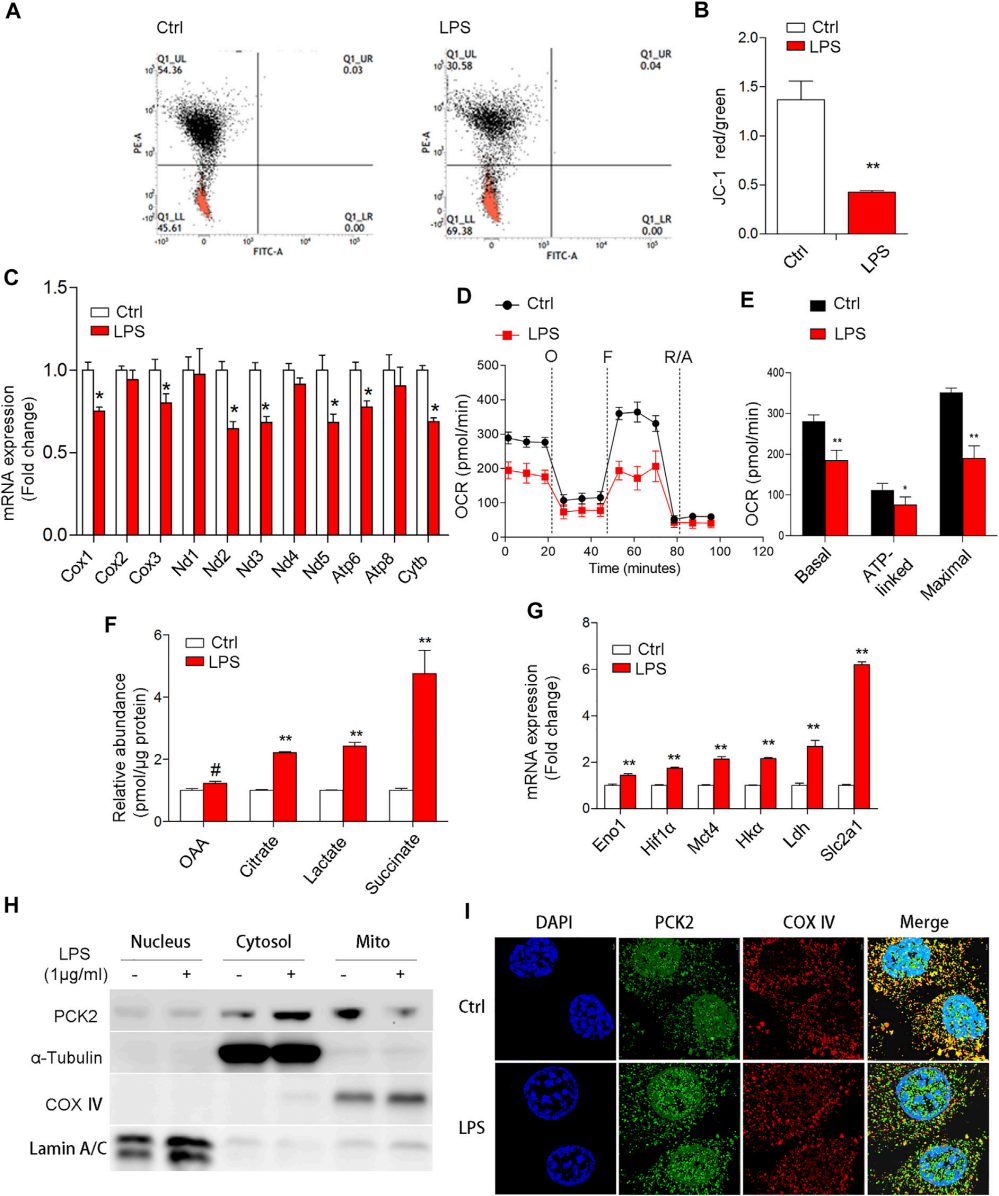
LPS-induced mitochondrial dysfunction and PCK2 aggregates in the cytosol but not mitochondria. **(A,B)** Mitochondrial membrane potential was measured using the JC-1 probe by flow cytometry. **(C)** The mRNA expression of mitochondrial DNA-encoded genes was measured by real-time PCR. **(D)** Oxygen consumption (OCR) was measured with a Seahorse XF-24 analyzer. **(E)** The quantification of basal OCR, ATP-linked OCR, and maximal OCR. **(F)** The contents of mitochondrial or glycolytic intermediates including OAA, citrate, lactate, and succinate were measured by HPLC. **(G)** The mRNA expression of glycolytic enzymes was measured by real-time PCR. **(H)** The distribution of PCK2 was measured by Western blot using isolated protein from nuclear, cytosolic, and mitochondrial fractions. **(I)** Double immunofluorescence staining of PCK2 and COX Ⅳ by confocal laser microscopy. Values are mean ± SEM. *p < 0.05, **p < 0.01.

Since PCK2 protein was not regulated at the transcription level, we presumed that upregulated PCK2 may be caused by mitochondrial dysfunction, which has been linked to the accumulation of proteins in the cytosol (Genini et al., 2017). Indeed, LPS stimulation reduced the mitochondrial membrane potential (ψm) and decreased the mRNA expression of mitochondrial DNA-encoded genes (Figures 3A–C), as well as the mitochondrial oxygen consumption rate (Figures 3D,E). Moreover, some intermediates that reflect a metabolic shift from mitochondrial OXPHOS to glycolysis in M1 macrophage, were determined by HPLC. The data showed that the production of citrate, succinate, oxaloacetate, and lactate were dramatically increased upon LPS stimulation (Figure 3F), which is in line with the mRNA expression of glycolytic enzymes (Figure 3G). Next, we determined the distribution of PCK2 by Western blot using isolated protein from nuclear, cytosolic, and mitochondrial fractions. Interestingly, we found that PCK2 was mainly localized in the cytosol, rather than in the mitochondria upon LPS stimulation (Figure 3H), which was further confirmed by confocal laser microscopy with double staining of PCK2 and COX Ⅳ (Figure 3I). These results indicate that the translocation of PCK2 from the cytosol to mitochondria was inhibited by LPS.

### Upregulated Mitochondrial Phosphoenolpyruvate Carboxykinase2 Promotes Inflammatory Responses in Kupffer Cells

**FIGURE 4:**
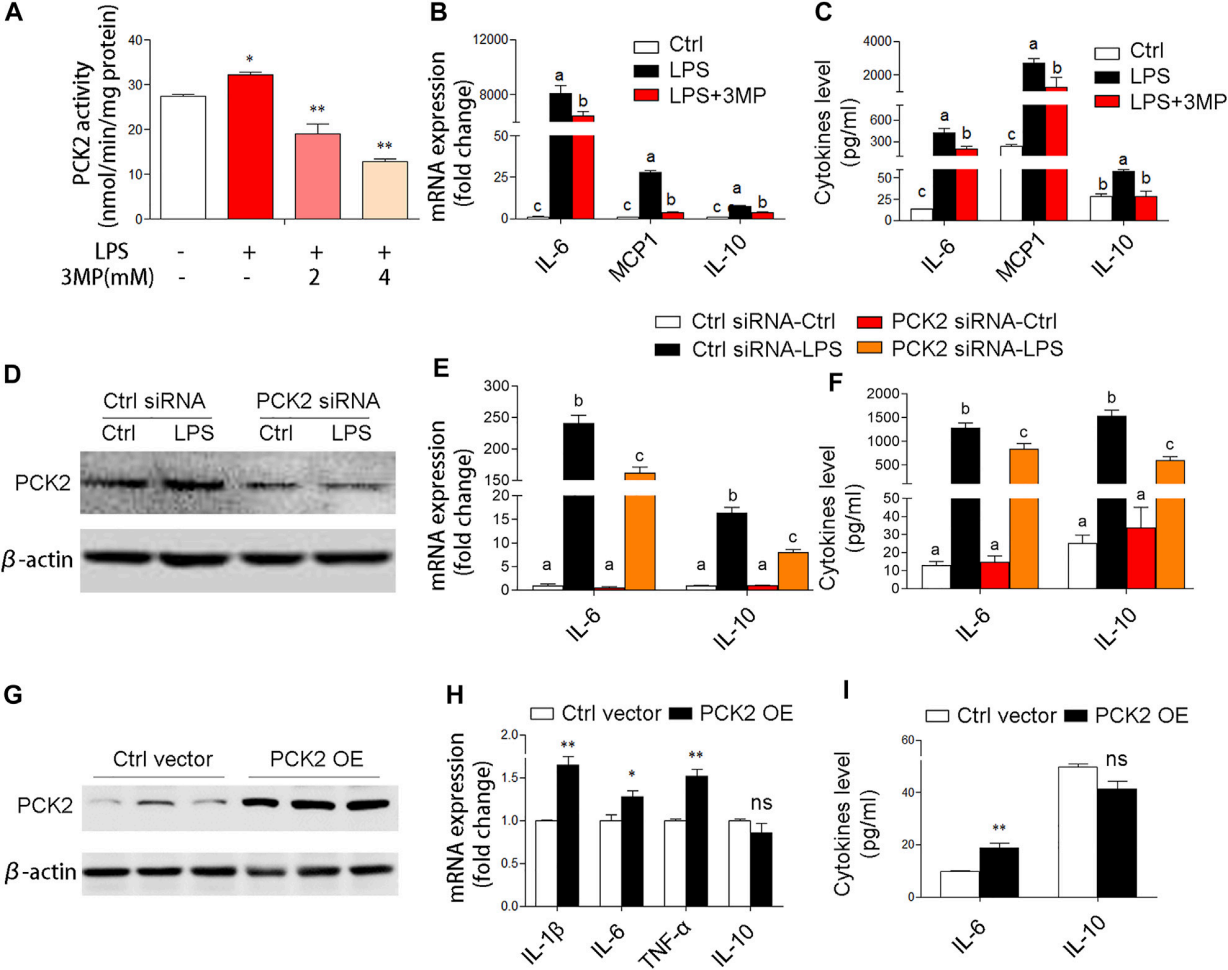
PCK2 promotes inflammatory responses in Kupffer cells. **(A)** The enzyme activity of PCK2 was measured after 3MP treatment in Kupffer cells. **(B)** The mRNA expression of cytokines including IL-6, MCP1, and IL-10 was measured by real-time PCR. **(C)** The production of cytokines in the medium was measured by cytometric bead array (CBA) after 3MP treatment. **(D)** The protein expression of PCK2 was determined by Western blot after siRNA knockdown. **(E)** The mRNA expression of IL-6 and IL-10 was measured by real-time PCR after PCK2 knockdown. **(F)** The production of cytokines was determined by CBA after PCK2 knockdown. **(G)** The protein expression of PCK2 was determined by Western blot after vector transfection. **(H)** The mRNA expression of cytokines including IL-1β, IL-6, TNFα, and IL-10 was measured by real-time PCR. **(I)** The production of cytokines was determined by CBA after PCK2 overexpression (PCK2 OE). Values are mean ± SEM. *p < 0.05, **p < 0.01. values with different superscript letters mean significant differences.

Based on the aforementioned observations, we decided to further investigate the potential role of PCK2 in inflammatory responses. Firstly, 3MP, which inhibits of PCK2, was used to block the function of PCK2. We found that 3MP strongly inhibited LPS-induced PCK2 activity in a dose-dependent manner (Figure 4A). After 3MP administration, LPS-induced cytokines expression and production such as IL-6, MCP1, and IL-10 were significantly decreased (Figures 4B,C). Similarly, siRNA-specific knockdown of PCK2 also reversed LPS-induced cytokines production (Figures 4D–F). Moreover, we overexpressed PCK2 in Kupffer cells and found that the mRNA expression of pro-inflammatory cytokines including IL-1β, IL-6, and TNFα was significantly upregulated. However, the expression of anti-inflammatory cytokine IL-10 was not altered (Figures 4G–I). These results suggest that upregulation of PCK2 promoted inflammation in Kupffer cells.

### Mitochondrial Phosphoenolpyruvate Carboxykinase2 Promotes Inflammation via Modulating Protein Phosphorylation of NF-κB and AKT/MAPK Signaling Pathways

**FIGURE 5:**
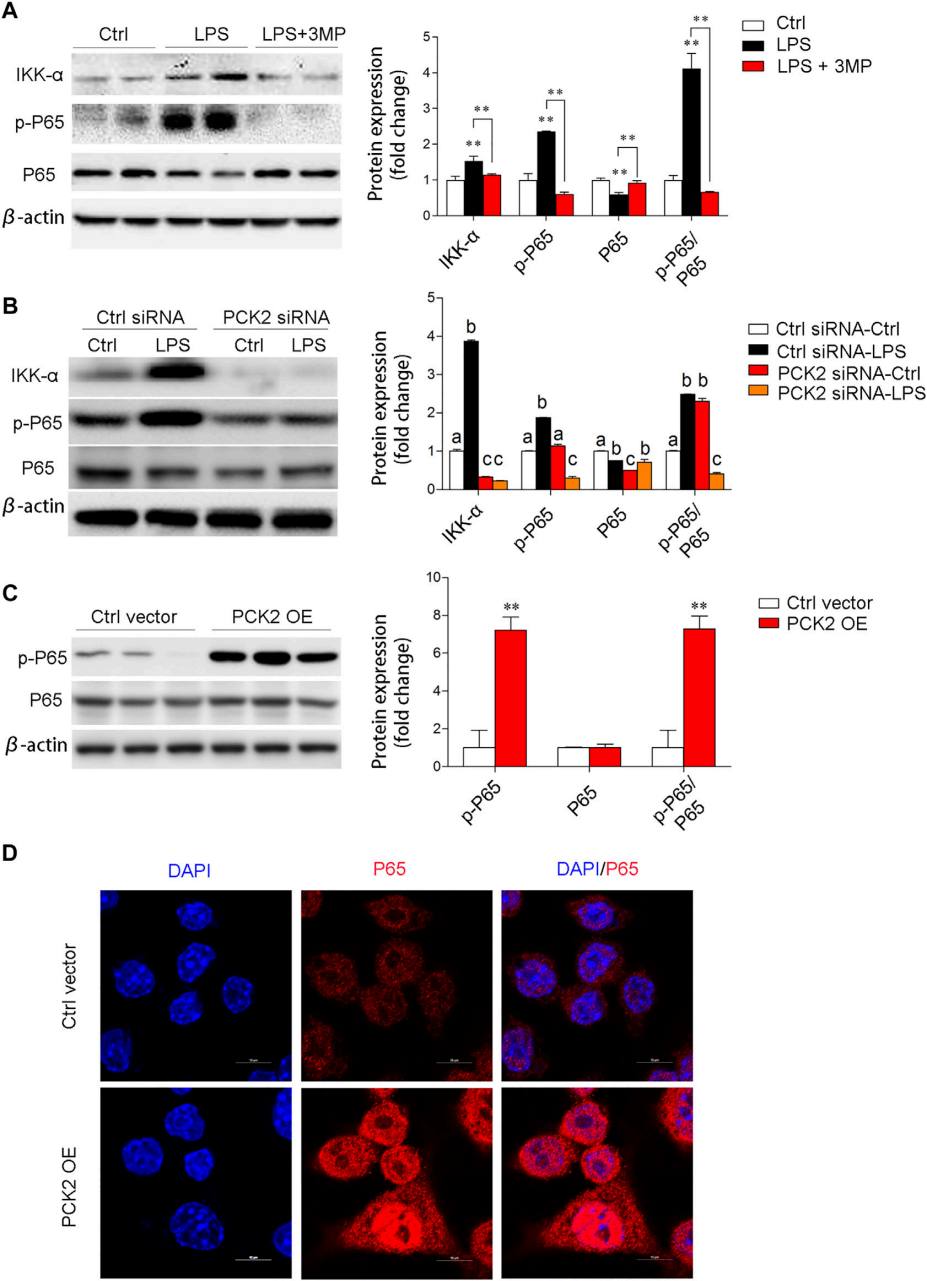
PCK2 promotes inflammation via regulating NF-κB activation. **(A)** The total protein expression of IKKα, p-p65, and p65 was determined by Western blot after 3MP treatment. **(B)** The total protein expression of IKKα, p-p65, and p65 was determined by Western blot after PCK2 knockdown. **(C)** The total protein expression of p-p65 and p65 were determined by Western blot after PCK2 overexpression (PCK2 OE). **(D)** Representative fluorescence images of p65 nuclear translocation in Kupffer cells after PCK2 overexpression (PCK2 OE). (scale bar: 10μm). Values are mean ± SEM. *p < 0.05, **p < 0.01.

To elucidate the potential mechanisms of PCK2 in the regulation of inflammation, we examined several signaling pathways associated with macrophage activation. Similarly, pharmacological inhibition, knockdown, or overexpression of PCK2 were performed. The transcription factor NF-κB is a master regulator of the inflammatory response and LPS-stimulated phosphorylation and nuclear translocation of p65 NF-κB have been well documented. In this study, we found that inhibition of PCK2 by its inhibitor or siRNA, significantly decreased phosphorylated p65 NF-κB as well as IKK α, which control the translocation of p65 NF-κB (Figures 5A,B). On the contrary, PCK2 overexpressed in Kupffer cells induced p65 NF-κB phosphorylation and nuclear translocation (Figures 5C,D).

**FIGURE 6:**
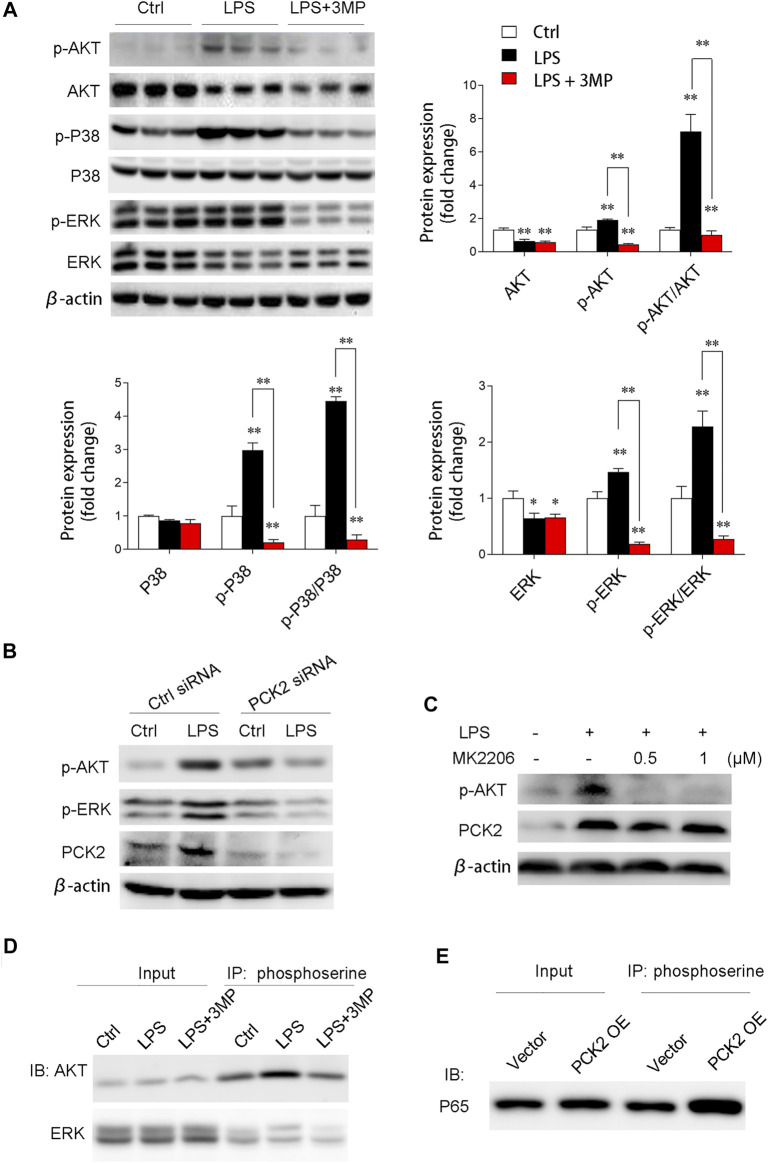
PCK2 promotes inflammation via modulating protein phosphorylation of NF-κB and AKT/MAPK signaling pathways. **(A)** Representative Western blots and quantification of p-AKT, AKT, p-p38, p38, p-ERK, and ERK. **(B)** Representative Western blots showing levels of p-AKT and p-ERK in Kupffer cells after PCK2 knockdown. **(C)** Representative Western blots showing levels of p-AKT and PCK2 in Kupffer cells after AKT inhibitor MK2206 treatment. **(D)** Phosphorylation of ERK and AKT were measured by immunoprecipitated (IP) with anti-phosphoserine and immunoblotted (IB) with anti-ERK and anti-AKT antibodies. **(E)** Phosphorylation of P65 NF-κB was measured by immunoprecipitated (IP) with anti-phosphoserine and immunoblotted (IB) with anti-P65 antibody. Values are mean ± SEM, *p < 0.05, **p < 0.01.

We then examined the AKT/MAPK (such as p38, ERK) signaling pathway, which has been shown to mediate NF-κB subunit p65 transcriptional activation and nuclear translocation (Madrid et al., 2001). The results demonstrated that phosphorylated AKT, p38, and ERK were significantly decreased after 3MP treatment (Figure 6A). Similarly, PCK2 knockdown by siRNA also decreased phosphorylated AKT and ERK (Figure 6B). To further prove that AKT/MAPK signaling was regulated by PCK2, an AKT-specific inhibitor, MK2206, was used to treat Kupffer cells under LPS stimulation. The data revealed that PCK2 was not altered by MK2206 while AKT signaling was blocked, indicating that PCK2 was an upstream molecule of the AKT/MAPK signaling pathway (Figure 6C). Next, to further determine the function of PCK2 in NF-κB activation and phosphorylation of AKT/MAPK pathways, co-immunoprecipitation (Co-IP) was performed. The results showed that the phosphorylation of serine protein AKT and ERK were suppressed by 3MP administration, while overexpression of PCK2 enhanced the phosphorylation of p65 NF-κB. (Figures 6D,E).

## Discussion

This study revealed that gluconeogenic enzyme PCK2 plays a non-metabolic role in the activation of Kupffer cells under LPS stimulation. Specifically, the upregulated PCK2 protein in Kupffer cells rather than hepatocytes were observed under LPS stimulation. In addition, we demonstrated that LPS-induced inflammation was attenuated by pharmacological inhibition or siRNA-mediated knockdown of PCK2. Moreover, we observed that pro-inflammatory genes, but not anti-inflammatory genes were significantly increased under the condition of PCK2 overexpression, thus, upregulation of PCK2 is an important mediator for inflammatory response. Mechanistic studies revealed that PCK2 mediated protein phosphorylation in NF-κB and AKT/MAPK- signaling pathways. Taken together, our results indicate that PCK2 is a key regulator in LPS-induced Kupffer cell activation.

During inflammation, macrophages play a crucial role in the initiation of inflammation and clearance of pathogens (Shapouri-Moghaddam et al., 2018). Kupffer cells are the resident macrophages in the liver, which play an essential role in innate immune response for LPS clearance (Su et al., 2000; Seki and Brenner, 2008; Dixon et al., 2013; Nguyen-Lefebvre and Horuzsko, 2015). The surface of Kupffer cells exhibits different TLRs including TLR2 and TLR4, which can recognize and bind to LPS. However, the roles of TLR2 and TLR4 as LPS receptors to induce immune activation are still controversial. Some studies conducted in TLR4 deficiency mice suggest that TLR4 strongly mediates LPS signaling, while other studies indicate that TLR2 can be induced in mouse macrophages in response to LPS stimulation (Du et al., 1999; Hoshino et al., 1999; Matsuguchi et al., 2000). In the present study, we found that acute LPS treatment induced a higher expression of TLR2, but not TLR4 at a dose of 5 mg/kg body weight after 2 h injection, which is consistent with our *in vitro* results. After activation, macrophages rapidly polarize from M0 to the M1 phenotype and secrete large amounts of pro-inflammatory cytokines and chemokines, which recruit more circulating neutrophils and monocytes to amplify the inflammatory response (Viola et al., 2019). In the present study, the ratio of CD68 and F4/80 positive Kupffer cells, and cytokines including IL-6, IL-10, MCP1, IFNγ, TNFα, IL10, and IL-12p70 were markedly increased after LPS stimulation, which confirmed that the pro-inflammatory phenotype of Kupffer cells was induced.

Interestingly, we found that the expression of PCK2 displayed different response to LPS in hepatocytes and Kupffer cells. PCK2 expression was reduced in hepatocytes but increased in Kupffer cells in response to LPS. Moreover, PCK2 accumulated in the cytosol, rather than in the mitochondria upon LPS stimulation in Kupffer cells. This differential expression and subcellular translocation may relate to LPS-induced body energy consumption such as glycogen depletion (Anders et al., 2016) and/or relate to the immune cells requiring energy for accelerating cytokine synthesis and release (Straub, 2017). During these biological processes, the metabolic shift has occurred. However, whether PCK2 plays a key role in metabolic shift needs further exploration.

Mitochondrial response to inflammation is crucial in the metabolic adaptation to infection. In M1 macrophages, a dramatic metabolic reprogramming happened, that is shifting from mitochondrial OXPHOS to cytosolic glycolysis (Ramond et al., 2019; Viola et al., 2019). During this process, several typical metabolic events occur in response to LPS such as succinate accumulation, lactate generation, and citrate transport from mitochondria (Palsson-Mcdermott et al., 2015; Wolf et al., 2016; Alves et al., 2020). Previous studies suggested that downregulation of PCK2 reduced mitochondrial OXPHOS, caused OAA accumulation (Tang et al., 2019), and promoted glycolysis in tumor cells (Chu et al., 2017; Luo et al., 2017). However, whether this phenomenon also exists in macrophages is not clear. In the present study, we found that along with PCK2 upregulation, the production of OAA, citrate, succinate, and lactate was increased in Kupffer cells under LPS stimulation. Moreover, the mRNA expressions of metabolic enzymes involved in glycolysis were significantly increased after LPS stimulation. These data indicate that the function of PCK2 in macrophages may differ from cancer cells.

Increasing evidence indicates that some metabolic enzymes perform non-canonical functions by changing their original intracellular distribution, such as transferring from the cytosol to the nucleus (Wang et al., 2014), mitochondria (Qi et al., 2019), or plasma membrane (Bae et al., 2012). For example, Shirai et *al.*(Shirai et al., 2016) and Yang et *al.*(Yang et al., 2015) found that a key enzyme of glycolysis, pyruvate kinase M2 (PKM2), could enter the nucleus and phosphorylate the transcription factor STAT3, thus boosting IL-6 and IL-1β production. PCK2 reaction is the only pathway of communicating mitochondrial intermediates with the glycolytic intermediary pool above phosphoenolpyruvate (Mendez-Lucas et al., 2014). Whether the subcellular localization of PCK2 is changed under LPS stimulation is unknown. In the present study, interestingly, we observed that upregulated PCK2 under inflammation was mainly localized in the cytosol, but not in mitochondria. It suggests that PCK2 translocation from cytosol to mitochondria was blocked under LPS stimulation. Therefore, we believed that in addition to its effect on OAA metabolism in the mitochondria, PCK2 could play an important role in immune activation of Kupffer cells.

AKT/MAPK-mediated NF-κB activation during LPS stimulation has been well documented (Vergadi et al., 2017; Dorrington and Fraser, 2019). In the present study, we also explored the potential effect of PCK2 on this signaling pathway. We found that the inflammatory responses such as genes expression and production of inflammatory cytokines were strongly decreased after 3MP treatment or siRNA knockdown. Correspondingly, PCK2 overexpression dramatically increased genes expression and production of pro-inflammatory cytokines as well as promoting NF-κB nuclear translocation. These data indicate that PCK2 promotes the activation of inflammatory response in Kupffer cells. Pharmacological inhibition or knockdown of PCK2 impairs protein phosphorylation of AKT/MAPK signaling pathway, which participates in modulating NF-κB phosphorylation and transactivation (Madrid et al., 2001; Nandipati et al., 2017). In addition, Co-IP analysis further confirmed the function of PCK2 in NF-κB activation and phosphorylation of AKT/MAPK pathways. Inhibition of PCK2 by 3MP downregulated phosphorylation level of serine residues on AKT. In contrast, overexpression of PCK2 upregulated the phosphorylation level of serine residues on p65 NF- κB.

**FIGURE 7:**
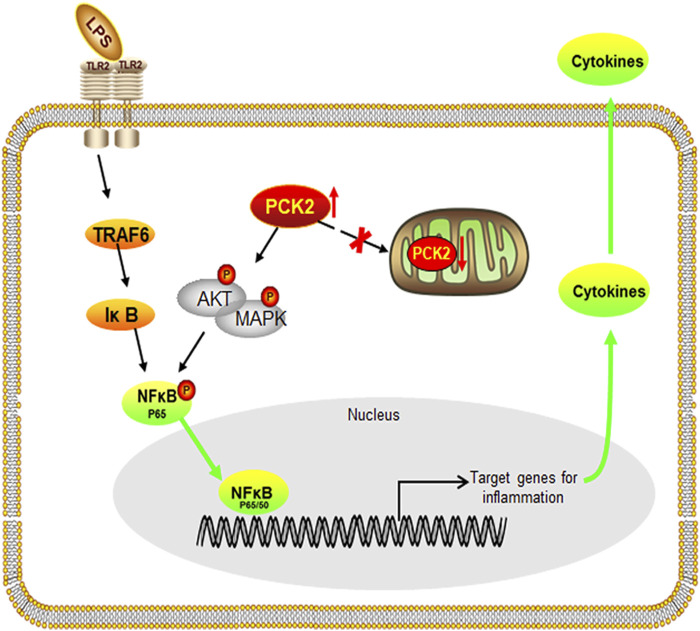
Schematic diagram of the potential mechanism and the effect of PCK2 on Kupffer cell activation. Upregulated PCK2 promotes LPS-induced inflammatory responses in Kupffer cells by regulating NF-κB activation and phosphorylation of AKT/MAPK.

In conclusion, our findings uncover the role of PCK2 in the regulation of inflammatory response in Kupffer cells. We have demonstrated that LPS stimulation results in PCK2 accumulation in the cytosol. Upregulated PCK2 exhibits a non-canonical role in Kupffer cells activation through modulating NF-κB translocation and protein phosphorylation (Figure 7), which provides a new therapeutic target for the regulation of Kupffer cells-mediated inflammatory responses.

## Data Availability

The raw data supporting the conclusions of this article will be made available by the authors, without undue reservation.

## Abbreviations

PCK2, mitochondrial phosphoenolpyruvate carboxykinase; CBA, cytometric bead array; ERK, extracellular signal-regulated kinase; NF-κB, nuclear factor kappa-light-chain-enhancer of activated B cells; AKT, RAC-alpha serine/threonine-protein kinase; p38, mitogen-activated protein kinase; IL, interleukin; TNF, tumor necrosis factor; MAPK, mitogen-activated protein kinase; TLRs, toll-like receptors; HPLC, High-performance liquid chromatography; PKM2, pyruvate kinase M2; ENO1, Enolase 1. OCR, Oxygen consumption rates; OE, overexpression.
