# Supplementary material for: A Novel Function of Mitochondrial Phosphoenolpyruvate Carboxykinase as a Regulator of Inflammatory Response in Kupffer Cells
Source: Front Cell Dev Biol. 2021 Dec 14;9:726931. doi: 10.3389/fcell.2021.726931 (PMC8712867; doi:10.3389/fcell.2021.726931)
Supplement: Supplementary file 1 [file DataSheet1.docx]

**Supplementary Information**

**A Novel Function of Mitochondrial Phosphoenolpyruvate Carboxykinase as a Regulator of Inflammatory Response in Kupffer Cells**

Haibo Dong^1,2^, Yue Feng^1,2^, Yang Yang^1,2^, Yun Hu^1,2^, Yimin Jia^1,2^, Shu Yang^1,2^, Nannan Zhao^1,2^, Ruqian Zhao^1,2*^

^1^MOE Joint International Research Laboratory of Animal Health & Food Safety, Nanjing Agricultural University, Nanjing, Jiangsu, China

^2^Key Laboratory of Animal Physiology & Biochemistry, Nanjing Agricultural University, Nanjing, Jiangsu, China

* Correspondence:

Ruqian Zhao,

Address: Key Laboratory of Animal Physiology & Biochemistry, College of Veterinary Medicine, Nanjing Agricultural University, Nanjing 210095, China.

Tel: 0086-25-84395047

Email: zhaoruqian@njau.edu.cn

**Table S1 Nucleotide sequences of primers**

| Gene name | Accession NO. | Forward primer  (5′-3′) | Reverse primer  (5′-3′) |
| --- | --- | --- | --- |
| **Inflammatory genes** | | | |
| TNFα | NM_013693.3 | GACGTGGAACTGGCAGAAGA | ACTGATGAGAGGGAGGCCAT |
| TLR-2 | NM_011905.3 | ATGTTGAAGTCCAGCAGAAT | CCGAACCAGGAGGAAGAT |
| TLR-4 | NM_021297.3 | TTCACCTCTGCCTTCACT | GGACTTCTCAACCTTCTCAA |
| NFκB(P65) | NM_009045.4 | GAAGCACAGATACCACCAA | CAGCCTCATAGTAGCCATC |
| IL-1β | NM_008361.4 | CTTCAGGCAGGCAGTATC | CAGCAGGTTATCATCATCATC |
| IL-10 | NM_010548.2 | CAGAGAAGCATGGCCCAGAA | GCTCCACTGCCTTGCTCTTA |
| IL-6 | NM_031168.2 | GCCTTCTTGGGACTGATGCT | GACAGGTCTGTTGGGAGTGG |
| IL12b | NM_001303244 | ATTACTCCGGACGGTTCACG | GCCATTCCACATGTCACTGC |
| MCP1 | NM_011333.3 | TCGGAACCAAATGAGATCAGA | TAGCTTCAGATTTACGGGTCA |
| F4/80 | [NM_010130.4](https://www.ncbi.nlm.nih.gov/entrez/viewer.fcgi?db=nucleotide&id=183583543) | TCTGGGGAGCTTACGATGGA | GAATCCCGCAATGATGGCAC |
| **Metabolic genes** | | | |
| Pck2 | NM_028994.2 | CTAGGCTAGCAAGAGCACAGC | TATTCGAGCCTTCGAGCCAA |
| Slc2a1 | XM_006502908.1 | ACCACGCTTTGGTCTCTCTC | GCCCAGGATCAGCATCTCAA |
| Hk2 | NM_013820.3 | TGATCGCCTGCTTATTCACGG | AACCGCCTAGAAATCTCCAGA |
| Ldhα | NM_001136069.2 | TCAGGCGGCTACACGTACA | GGAGATCCATCATCTCGCC |
| Eno1 | NM_023119.2 | GAGGTCGATCTGTACACCGC | CTAACCAGAGCAGGCGCAAT |
| Hif1a | NM_001313920.1 | TGACGGCGACATGGTTTACA | AATATGGCCCGTGCAGTGAA |
| **Mitochondrial DNA-encoded genes** | | | |
| Cox1 | AP014541.1 | GACTTGCAACCCTACACGGA | GATGGCGAAGTGGGCTTTTG |
| Cox2 | AP014541.1 | ATAACCGAGTCGTTCTGCCA | CCTGGTCGGTTTGATGCTACT |
| Cox3 | AP014541.1 | TAACTGGAGCCTTTTCAGCCC | AATAGGAGTGTGGTGGCCTTG |
| Nd1 | AP014541.1 | TCCTATCCACGCTTCCGCTA | GGTGGTATCCCCGCTGTAAA |
| Nd2 | AP014541.1 | ATCCTCCTGGCCATCGTACT | ATCAGAAGTGGAATGGGGCG |
| Nd3 | AP014541.1 | TGCACGCCTACCATTCTCAA | GGTAGTGGAAGTAGAAGGGCA |
| Nd4 | AP014541.1 | TAATCGCACATGGCCTCACA | CATTTGAAGTCCTCGGGCCA |
| Nd5 | AP014541.1 | GCAGCCCTACAAGCAATCCT | GTCATGGGTGGAGGCCAAAT |
| Atp6 | AP014541.1 | ACACACCAAAAGGACGGACA | AGTGGGCGAGTGAGCTTTTT |
| Atp8 | AP014541.1 | ACAAACATTCCCACTAGCACCT | GTTGGGGTAATGAATGAGGCAAA |
| Cytb | AP014541.1 | TTTATCATCGCGGCCCTAGC | ATGGGGTGGGGTGTTTAGTG |
| **Reference gene** | | | |
| RPS17 | NM_009092.3 | GGAGATCGCCATTATCCCCA | ATCTCCTTGGTGTCGGGATC |

# Table S2 Body weight, liver weight, liver index and biochemical parameters of blood plasma after 2 h LPS injection.

| **Parameters** | **Ctrl** | **LPS** |
| --- | --- | --- |
| Liver | | |
| Body weight (g) | 20.77 ± 0.24 | 20.71 ± 0.24 |
| Liver weight (g) | 1.20 ± 0.02 | 1.18 ± 0.04 |
| Liver index (%) | 5.76 ± 0.09 | 5.68 ± 0.19 |
| Plasma | | |
| ALT (U/L) | 9.25 ± 1.31 | 16.80 ± 3.15 (*P* = 0.06) |
| AST (U/L) | 68.50 ± 3.70 | 82.30 ± 6.32 (*P* = 0.08) |
| Glucose (mmol/L) | 7.44 ± 0.27 | 5.46 ± 0.45 ^**^ |
| LDH (U/L) | 229 ± 13 | 357 ± 27 ^**^ |

Values are mean ± SEM, **P < 0.01.


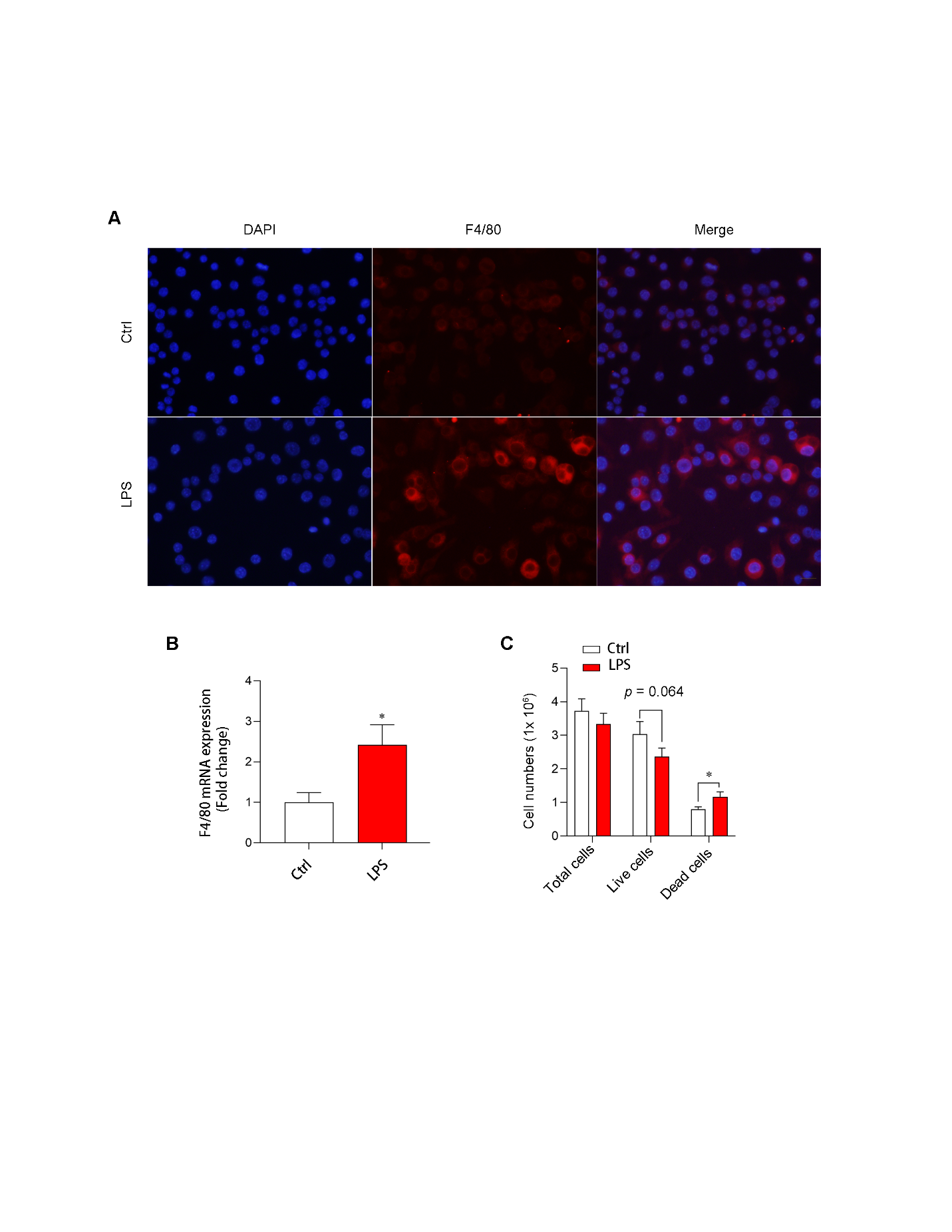


**Figure S1** Effects of LPS on Kupffer cells proliferation. (**A**) Immunofluorescence microscopy of F4/80 (565409, BD biosciences) with LPS stimulation for 12 h in Kupffer cells. Scale Bar = 20 μm. (**B**) mRNA expression of F4/80 after LPS stimulation for 12 h in Kupffer cells. (**C**) Cell number counting by an automated cell counter (Invitrogen C10281 Countess). Values are mean ± SEM, *P < 0.05.


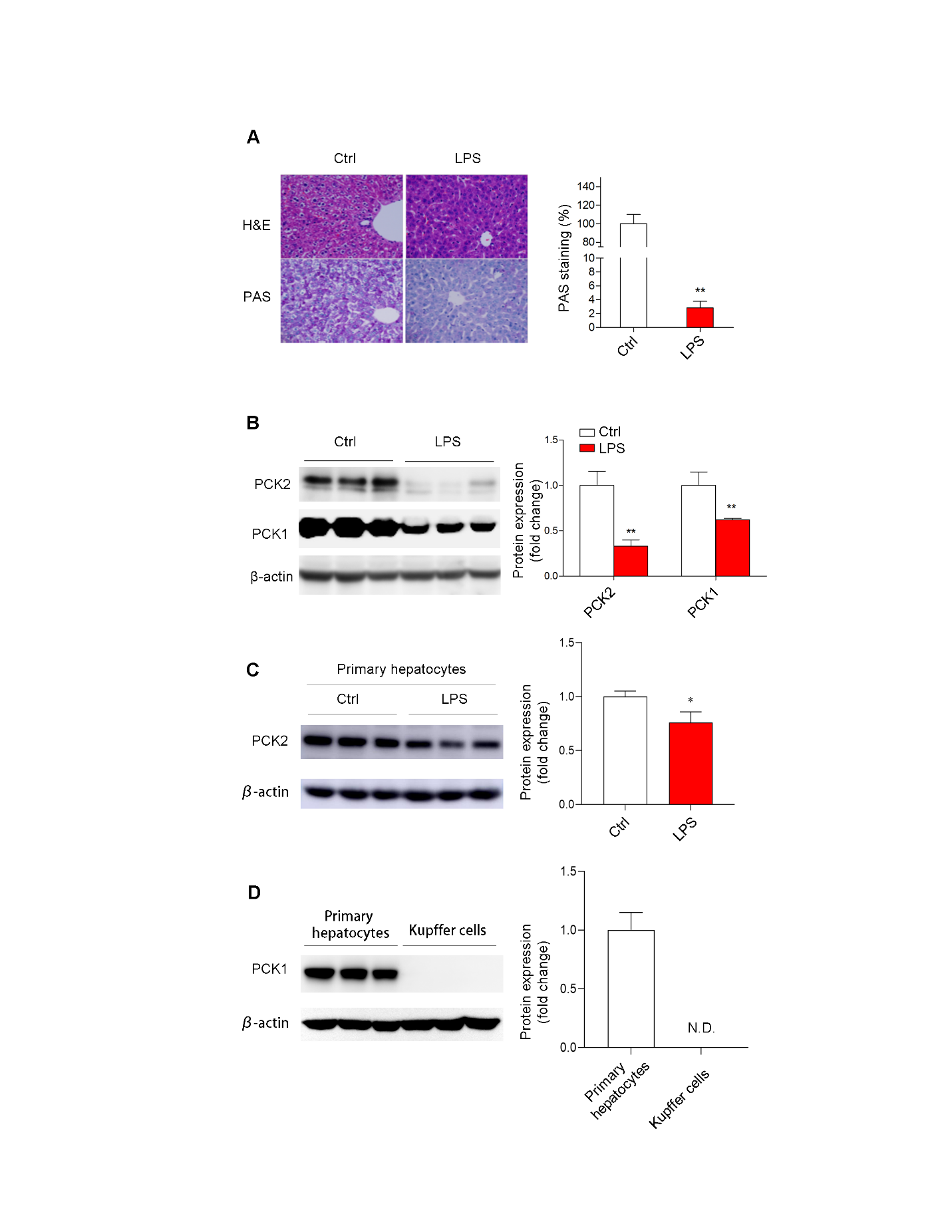


**Figure S2** Effects of LPS on liver injury, hepatic glycogen consumption and expression of hepatic gluconeogenic enzymes. (**A**) H&E and PAS staining. (**B**) The protein expression of gluconeogenic enzymes including PCK1 and PCK2 were determined in liver by Western blot. (**C**) The protein expression of PCK2 was performed by Western blot in primary hepatocytes after 12 h LPS treatment. (**D**) Identification of PCK1 expression in primary hepatocytes and Kupffer cells by Western blot. Values are mean ± SEM, *P < 0.05, **P < 0.01, N.D.= not detectable.


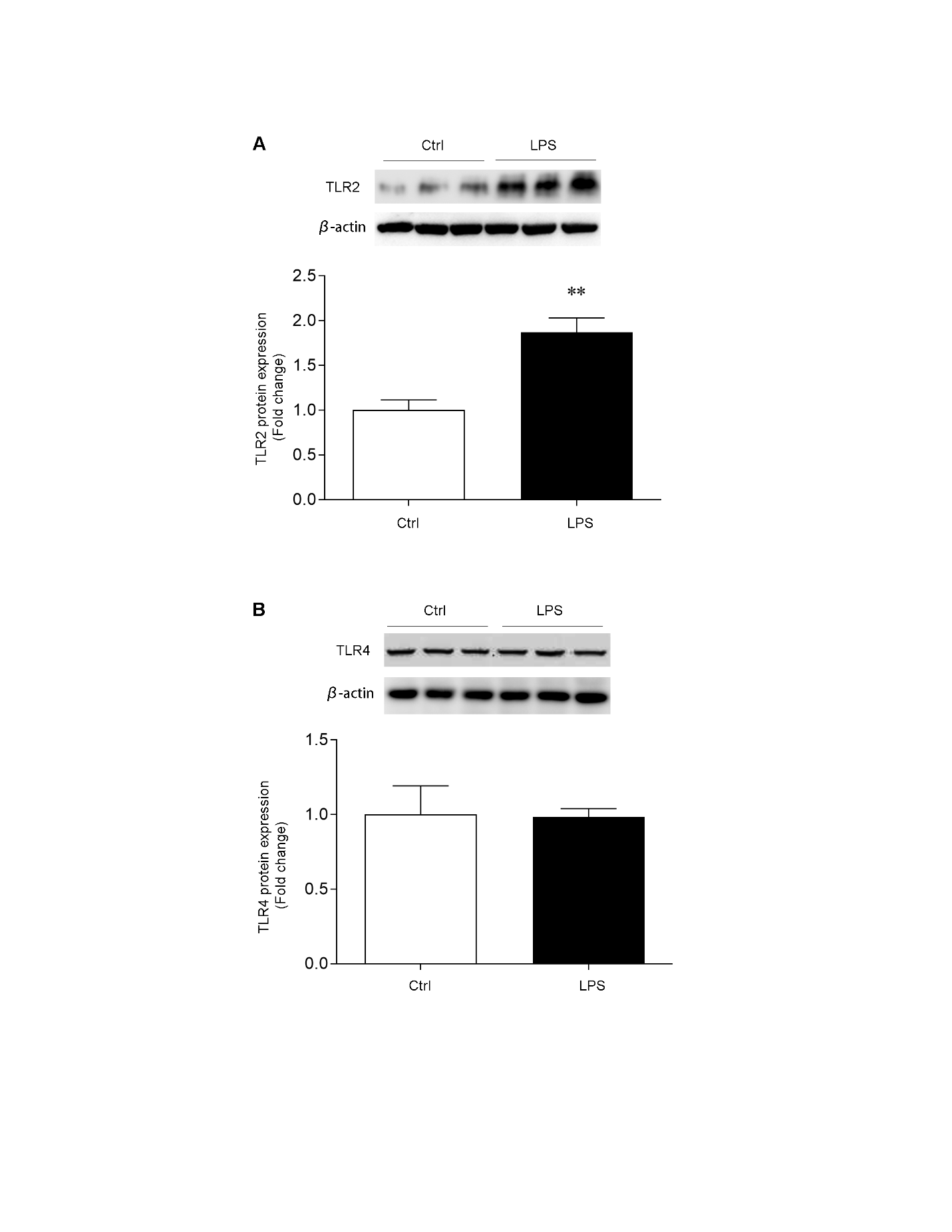


**Figure S3** Effects of LPS on TLR2 and TLR4 expression. (**A**) The protein expression of TLR2 was determined by Western blot in Kupffer cells after 12 h LPS stimulation. (**B**) The protein expression of TLR4 was determined by Western blot in Kupffer cells after 12 h LPS stimulation. Values are mean ± SEM, **P < 0.01.
